# Perceived social support and self-stigma as factors of COVID-19 booster vaccination behavior and intention via cognitive coping and emotion regulation among people infected with COVID-19 in Hong Kong

**DOI:** 10.1186/s12889-025-21899-x

**Published:** 2025-02-18

**Authors:** Xiaoying Zhang, Yiming Luan, Yihan Tang, Mason M. C. Lau, Yanqiu Yu, Jing Gu, Joseph T. F. Lau

**Affiliations:** 1 College of Public Health, Shanghai University of Medicine and Health Sciences, Shanghai, 201318 China; 2 Meilong Community Health Center of Minhang District, Shanghai, 201100 China; 3 School of Public Health, Sun Yat-Sen University, Guangzhou, 510080 China; 4 Jockey Club School of Public Health and Primary Care, The Chinese University of Hong Kong, Hong Kong, China; 5 School of Public Health, Fudan University, Shanghai, 200032 China; 6 Public Mental Health Center, School of Mental Health, Wenzhou Medical University, Wenzhou, 325000 China; 7 Zhejiang Provincial Clinical Research Center for Mental Disorders, The Affiliated Wenzhou Kangning Hospital, Wenzhou Medical University, Wenzhou, 325000 China; 8 Center for Health Behaviors Research, The Chinese University of Hong Kong, Hong Kong, China

**Keywords:** Booster COVID-19 vaccines, Perceived social support, Self-stigma, Rumination, Catastrophizing, Active coping, Mediation, Stress coping model, Emotion regulation

## Abstract

**Background:**

It is imperative to promote behavior/intention of taking up booster COVID-19 vaccination (BI-BV) among people who have ever contracted COVID-19 (PECC). The aims were to investigate the prevalence of BI-BV and its associations with perceived social support. Guided by the stress coping theory, we tested mediators between perceived social support and BI-BV via self-stigma, active coping, and maladaptive emotion regulation (rumination and catastrophizing).

**Methods:**

A random population-based telephone survey was conducted among adult PECC having completed the primary series of COVID-19 vaccination prior to the diagnosis; 230 participants were interviewed from June to August 2022 during the fifth (last) major outbreak in Hong Kong. The associations between the independent variables and BI-BV were tested by logistic regression analysis. A structural equation model (SEM) tested the indirect effects of the latent variables of self-stigma, active coping, and maladaptive emotion regulation between the latent variable of perceived social support and BI-BV.

**Results:**

The prevalence of BI-BV was 62.2%. It was associated with age, marital status, full-time employment, and chronic disease status. The logistic regression analysis found that BI-BV was positively associated with perceived social support (ORc = 1.31, 95% CI: 1.12– 1.54), active coping (ORc = 1.40, 95% CI: 1.10– 1.79), rumination (ORc = 1.75, 95% CI: 1.13– 2.70), and catastrophizing (ORc = 3.12, 95% CI: 1.49– 6.51) and negatively associated with self-stigma (ORc = 0.80, 95% CI: 0.72– 0.88). In the SEM analysis, the positive association between perceived social support and BI-BV was fully mediated: 1) via self-stigma (β = 0.07, 95% CI: 0.03– 0.14), 2) via active coping (β = 0.06, 95% CI: 0.02– 0.12), and 3) via self-stigma and then active coping (β = 0.01, 95% CI: 0.002– 0.04). Two of these indirect paths involved active coping. The indirect paths involving maladaptive emotion regulation were all non-significant.

**Conclusions:**

Perceived social support was associated with BI-BV, and was mediated via self-stigma, active coping, and serially self-stigma then active coping but not emotion maladaptation. The data supported the stress cognitive coping model in explaining the association between perceived social support and BI-BV. Interventions promoting BI-BV may consider modifying the observed significant factors. Future longitudinal studies are warranted to confirm the findings.

**Supplementary Information:**

The online version contains supplementary material available at 10.1186/s12889-025-21899-x.

## Introduction

Although COVID-19 is no longer a pandemic, it remains globally prevalent. The prevalence of COVID-19 reinfection was 28% when the BQ.1/BQ.1.1 variants were involved [1]. Reinfection increases the risks of death, hospitalization [2], and severity of long COVID symptoms (e.g., lung scarring, heart diseases, neurological effects, and psychological consequences) [3]. Booster vaccination can increase immunity against reinfection [4, 5]. According to a meta-analysis of 91 studies that investigated 158,478 records, the risk of reinfection after > = 12 months from the first infection was lower among vaccinated individuals than unvaccinated individuals, although the reinfection rates were low in both groups [6]. The World Health Organization and some country authorities recommend people to take up booster COVID-19 vaccination six months after diagnosis [7–9]. It is important to promote booster vaccination in specific sub-populations (e.g., older people). Vaccinated people who have ever been infected with COVID-19 (PECC) is a special population that warrants attention. The infection experience of the PECC might have affected their confidence in the vaccines whereas such confidence was a factor of vaccination [10]. The experience of COVID-19 infection may also modify perceptions on risk of infection, consequences of infection, and perceived efficacy of vaccination, which were potential determinants of booster vaccination [11]. According to social marketing principles, segmentation and tailored strategies would improve effectiveness of health promotion [12]. Identification of psychosocial factors of behavior/intention of taking up booster COVID-19 vaccination (BI-BV) among PECC is hence essential. One study conducted in Italy reported that female sex, negative affective state, and lower perceived risk were associated with reluctance to take up the vaccines after contracting COVID-19 [13]. Another study conducted in Hong Kong explored some psychological factors associated with BI-BV among PECC [14]. No other similar studies were found in literature.

The stress coping theory was used as this study’s framework [15]. It postulates that when facing a stressor, an individual’s coping resources would alleviate the level of stress and affect his/her coping strategies, which would in turn affect his/her coping responses and coping outcomes [15]. In this study, perceived social support was considered as a coping resource, self-stigma as a stressor related to COVID-19, active coping as a cognitive coping strategy, and BI-BV as a coping response. In literature, perceived social support is a coping resource [15, 16] that affects health behaviors (e.g., physical exercise, dietary behavior) [17–19], and COVID-19 vaccination [20, 21]; it was also associated with various types of coping strategies [22, 23], including active coping [24]. However, the association between social support and booster vaccination among vaccinated PECC remains unclear. Public stigma was common during the COVID-19 pandemic [25, 26] and the SARS outbreak [27]. Self-stigma related to COVID-19 refers to internalization of the public stigma [28]. It is a prevalent stressor which was positively associated with mental distress [29, 30], and was also negatively associated with COVID-19 vaccination intention/behavior [31] and active coping in previous studies [32]. There is a dearth of studies looking at the relationship between self-stigma and booster vaccination behavior/intention among vaccinated PECC.

Emotion regulation and coping strategies are closely related but distinctive constructs that evolve separately [33]; they involve different strategies (e.g., active coping for coping and rumination for emotion regulation) [34, 35] and measurement scales [36, 37]. Emotion regulation refers to how an individual respond to emotionally arousing situations; such regulation may be adaptive or maladaptive, leading to better or worse emotional states, respectively [38]. Coping refers to how an individual manages specific demands arising from stressful situations, which is not limited to emotional difficulties and is hence more general than emotion regulation [37]. Coping is usually a volitional and controlled process while emotion regulation can be automated or uncontrolled [39]. Furthermore, some researchers suggested that emotion regulation is a subset of coping (emotional coping) [40]; others highlighted their differences [41]; a few studies even found minimal or non-significant associations between the two [42]. Since both processes involve regulations under stressful situations [39], the present study integrated both coping (active coping) and maladaptive emotion regulation (rumination and catastrophizing) into the stress-coping framework. It contends that the associations between perceived social support/self-stigma and BI-BV would be mediated through both a ‘cognitive coping process’ (i.e., via active coping) and an ‘emotion regulation process’ (i.e., via maladaptive emotion regulation). Maladaptive emotion regulation is a subset of emotion regulation which may lead to harmful/ineffective consequences [43]. Rumination (how people concentrate on the ideas and emotions connected to unfavorable events) and catastrophizing (exaggerations of the seriousness of an event) are two common modes of maladaptive emotion regulation [37]. As previous studies found that perceived social support was associated with self-stigma [44–46] whereas emotion regulation was associated with coping strategies [47, 48], it is plausible that self-stigma, emotion regulation, and active coping would mediate between perceived social support and BI-BV serially. The hypothetical model is presented in Supplementary Fig. 1.

The study was conducted in Hong Kong, where various control measures against COVID-19 (e.g., social distancing, facemask use and provision of free COVID-19 vaccination) were implemented since February 2020 [49]. As of December 31, 2021, the total number of COVID-19 cases and deaths was about 12,649 and 213, respectively [50]. The severe fifth-wave outbreak occurred from late December, 2021 to January, 2023 [51], during which about 1.4 million new cases and about 13,000 deaths were reported [52]. This study was conducted from June 20 to August 2, 2022, hence during an acute phase of the COVID-19 pandemic.

This study investigated the prevalence of BI-BV among PECC in Hong Kong, China, and tested the significance of some potential factors of BI-BV, i.e., perceived social support, self-stigma related to COVID-19 infection, active coping, and maladaptive emotion regulation (rumination and catastrophizing). Using structural equation model (SEM) analysis, two single-mediator paths between the latent variable perceived social support and BI-BV via the latent variables of active coping/maladaptive emotion regulation and two others between the latent variable of self-stigma and BI-BV via the latent variables of active coping/maladaptive emotion regulation were tested. Three additional serial mediation paths were tested: a) perceived social support → self-stigma → active coping → BI-BV, b) perceived social support → self-stigma → maladaptive emotion regulation → BI-BV), and c) perceived social support → self-stigma → maladaptive emotion regulation → active coping → BI-BV (see Supplementary Fig. 1). To our knowledge, no previous studies have tested such mediation paths.

## Methods

### Study design

A cross-sectional random, population-based telephone survey was conducted in Hong Kong, China from June 20 to August 2, 2022.

### Participants

Inclusion criteria included: (1) Chinese aged 18 years or older, (2) PECC status determined by the screening question: “Have you been tested positive for COVID-19 via the Nucleic Acid Amplification Test (NAAT) or Rapid Antigen Test (RAT)? (Yes/No response options)”, (3) completion of the primary series of vaccination (at least two doses of the COVID-19 vaccines) prior to the COVID-19 diagnosis. Regarding exclusion criteria, as the epidemiology, social context, and experience of COVID-19 infection was changing over time [10], factors of BI-BV were time-dependent. Thus, exclusion criteria were: 1) PECC not being diagnosed during the fifth (last) major COVID-19 outbreak wave in Hong Kong, i.e., excluding diagnosis made prior to February l, 2022, 2) those having taken up four doses of vaccines prior to the diagnosis as free fifth dose vaccination was not then available in Hong Kong, 3) those having difficulties in understanding and/or responding to the questions according to the judgement of the trained interviewers, and 4) non Chinese speakers.

### Data collection

Telephone numbers were randomly generated from a fixed-line phone directory. Interviews, which took about 20 min to complete, were conducted between 5 and 10 pm to avoid oversampling of unemployed people. The eligible household member whose birthday was closest to the interview date was invited to participate in the study. At least three attempts were made to follow on the unanswered phone calls before classifying them as invalid numbers. The interviews were conducted in Chinese. Briefing was provided by the well-trained interviewers to ensure participants were informed about the anonymous and voluntary nature of the study. Verbal informed consent was obtained from the participants. The interviewers signed a form declaring that they had gone through the proper briefing/consent procedures. Previous studies have used similar methodologies [14, 53, 54]. No incentives were given to the participants.

A total of 469 valid telephone contacts were made, of which 280 participants completed the interview (response rate: 280/469 = 59.7%); 50 cases were excluded from data analysis (incompletion of primary series [*n* = 41], diagnosed before February 1, 2022 [*n* = 3], having taken up four doses of vaccines prior to the COVID-19 diagnosis [*n* = 1], and no response to the question about vaccination behavior/intention [*n* = 5]). The final effective sample size was 230.

### Measures

#### Background factors

Background information was collected (sex, age, education level, marital status, employment status, whether living alone, whether having a religious belief, and chronic disease status). Regarding chronic disease status, participants were asked: “Do you have the following chronic diseases (hypertension, diabetes, heart disease, lung disease, kidney disease, liver disease, respiratory disease, cancer, others)?” The response categories for each item were “yes”, “no”, “don’t know”, or “refuse to answer”. Those having at least one “yes” answer was classified as having one or more types of chronic disease.

#### Behavior/intention of taking up booster dose of COVID-19 vaccination (BI-BV)

It was assessed by the item: “After receiving your COVID-19 diagnosis, have you taken up a booster dose of COVID-19 vaccination or would you do so in the future? (1: neither having taken up nor intended to take up the booster vaccine; 2: having taken up the booster vaccine after the COVID-19 diagnosis; 3: had not taken up the booster vaccine but intended to do so).The three responses were recoded into a binary variable by combining 2) and 3) into the ‘individuals with either booster vaccination behavior or intention group’ (i.e., those with BI-BV) and the ‘individuals with neither booster vaccination behavior nor intention group’ (i.e., those without BI-BV).

#### Perceived social support

Perceived social support was assessed in two dimensions (perceived emotional support and perceived instrumental support), which have frequently been used to assess the level of perceived social support [55]. The two questions were: “Your family and friends would be available when you need emotional support or need someone to talk to” and “When you need practical help in life (such as financial needs), your family and friends will give you enough support”. Participants rated the items on two Likert scales, ranging from 1 (highly disagree) to 7 (highly agree). Previous studies have used similar questions to assess perceived social support [56, 57]. In this study, the Cronbach’s alpha of the scale was 0.85. A summation score of the two items was used to represent the level of perceived social support in the univariate logistic regression analysis.

#### Self-stigma related to COVID-19 infection

COVID-19-related self-stigma was assessed by using a 3-item scale [58]. The three items assessed the levels of reluctance to disclose COVID-19 infection to others, perceived negative views from others because of his/her COVID-19 infection status, and perceived public discrimination toward people infected with COVID-19. Likert scales were used to rate the item responses (1 = highly disagree to 5 = highly agree). In this study, the Cronbach’s alpha of the scale was 0.96. A summation score of the three items was used to represent the level of self-stigma in the univariate logistic regression analysis.

#### Active coping

Active coping was assessed by using the 2-item subscale of the Brief Coping Orientation to Problems Experienced (brief COPE) inventory [36]. The two items were “I have been concentrating my efforts on doing something about my situation” and “I have been taking actions to try to improve my situation”. The subscale has been used in COVID-19 related research [59, 60]. The items were rated by 4-point Likert scales, ranging from 1 = “I haven’t been doing this at all” to 4 = “I have been doing this a lot”. In this study, the Cronbach’s alpha of the scale was 0.94. A score summing up the two item scores was used in the univariate logistic regression analysis.

#### Maladaptive emotion regulation

Maladaptive emotion regulation was assessed by the rumination and catastrophizing subscales of the Cognitive Emotion Regulation Questionnaire-Short Version (CERQ-short) [37]. The two subscales have been used to investigate COVID-19 related maladaptive emotion regulations [61, 62]. Each of them has two items (“You constantly recall the pandemic as well as your feelings about the pandemic”, and “You indulge in your thoughts and feelings about the pandemic” for rumination, and “You constantly think about how terrible the pandemic is” and “Sometimes you feel like the disaster is coming” for catastrophizing). Each item was assessed on a 5-point Likert scale from 1 (never) to 5 (always). The two subscales were formed by summing up their two respective item scores. They were used in the univariate logistic regression analysis. In this study, the Cronbach’s alphas of the rumination and catastrophizing subscales were 0.97 and 0.81, respectively.

### Statistical analysis

BI-BV was used as the binary dependent variable. Univariable logistic regression analysis was performed to examine the individual associations between the background variables/independent variables (perceived social support, self-stigma, active coping, rumination, and catastrophizing) and BI-BV. Crude odds ratios (ORc) and respective 95% confidence intervals (CIs) were reported. The confidence level of 95% was used to define statistical significance.

A structural equation model (SEM) was fit to assess whether self-stigma, active coping, and maladaptive emotion regulation would mediate between perceived social support and BI-BV, using the weighted least squared mean and variance adjusted (WLSMV) estimator [63]. Three latent variables were constructed for perceived social support, self-stigma, active coping, whereas maladaptive emotion regulation was a second-order latent variable based on the rumination and catastrophizing subscales [64]. Satisfactory model fit indices included χ2/df < 5, comparative fit index (CFI) ≥ 0.90, Tucker-Lewis index (TLI) ≥ 0.90, and root mean square error of approximation (RMSEA) ≤ 0.08 [65]. The bootstrapping method (*n* = 2000) was conducted to test the significance of the indirect effects. An indirect effect was statistically significant when its 95% CI did not include zero. The SEM analysis was performed using Mplus 8.3, while the other tests were conducted using Stata 17.0 (Stata Corp, College Station, TX, USA).

## Results

### Participants’ characteristics

Sixty-three percent of the participants were female; 60.9% aged between 31–60 years; 92.6% did not live alone; 70.9% were currently married; 26.1% had received college or above education; 53.0% worked fulltime. Furthermore, 77.8% did not have a religious belief; 71.7% reported no chronic diseases. The prevalence of BI-BV was 62.2%, with 19 participants (8.3%) having taken up a booster vaccine after their COVID-19 diagnosis and 124 participants (53.9%) not having taken up the booster vaccines since diagnosis but intended to do so. The mean score was 5.70 for the self-stigma scale (SD = 2.97; range = 3–15), 11.18 for the perceived social support scale (SD = 1.80; range = 2–14), 6.69 for the active coping subscale (SD = 1.13; range = 2–8), 2.87 for the rumination subscale (SD = 1.43; range = 2–10), and 2.55 for the catastrophizing subscale (SD = 1.16; range = 2–10), respectively. The data are presented in Table 1.

**Table 1:** Participants’ characteristics (*n* = 230)

	n	%
**Sex**		
Female	145	63.0
Male	85	37.0
**Age**		
18–30	26	11.3
31–60	140	60.9
> 60	64	27.8
**Education level**		
< College	167	72.6
≧College	60	26.1
Missing data	3	1.3
**Current marital status**		
*Currently unmarried	67	29.1
Currently married	163	70.9
**Employment status**		
Fulltime	122	53.0
Part time	25	10.9
Others (retired, homemakers, students)	83	36.1
**Whether living alone**		
No	213	92.6
Yes	17	7.4
**Whether having religious belief**		
No	179	77.8
Yes	51	22.2
**Chronic disease status**		
No	165	71.7
Yes	65	28.3
**Behavior/intention of taking up booster dose of COVID-19 vaccination (BI-BV)**		
No	87	37.8
Yes	143	62.2

^*^Currently unmarried individuals included those who were unmarried, separated/divorced, cohabiting, widowed, and others

### Factors of BI-BV

People of older age (> 60 versus 18–30: ORc = 8.33, 95% CI: 2.92**–**23.81), being currently married (versus currently unmarried: ORc = 1.80, 95% CI: 1.01**–** 3.20), not engaged in fulltime work (part-time versus fulltime: ORc = 2.97, 95% CI: 1.11**–** 7.93; others versus fulltime: ORc = 2.60, 95% CI: 1.42**–** 4.75), and having chronic disease(s) (versus no/don’t know: ORc = 2.58, 95% CI: 1.34**–** 4.96) were more likely than others to have reported BI-BV. The factors of sex, education level, whether living alone, and religious belief were statistically non-significant. Positive associations with BI-BV were found for the factors of perceived social support (ORc = 1.31, 95% CI: 1.12**–** 1.54), active coping (ORc = 1.40, 95% CI: 1.10**–** 1.79), rumination (ORc = 1.75, 95% CI: 1.13 2.70), and catastrophizing (ORc = 3.12, 95% CI: 1.49**–** 6.51). A negative association was found for the factor of self-stigma (ORc = 0.80, 95% CI: 0.72**–** 0.88). The results are presented in Table 2.

**Table 2:** Factors of behavior/intention of taking up booster COVID-19 vaccination

	ORc (95% CI)
**Sex**	
Female	Reference = 1.0
Male	0.80 (0.45**–** 1.38)
**Age**	
18–30	Reference = 1.0
31–60	1.67 (0.72**–** 3.88)
> 60	8.33 (2.92**–** 23.81)
**Educational level**	
< College	Reference = 1.0
≧College	0.67 (0.37**–** 1.22)
Missing data	1.09 (0.10**–** 12.30)
**Current marital status**	
*Currently unmarried	Reference = 1.0
Currently married	1.80 (1.01**–** 3.20)
**Employment status**	
Fulltime	Reference = 1.0
Part-time	2.97 (1.11**–** 7.93)
Others (retired, homemakers, students)	2.60 (1.42**–** 4.75)
**Whether living alone**	
No	Reference = 1.0
Yes	1.50 (0.51**–** 4.42)
**Whether having a religious belief**	
No	Reference = 1.0
Yes	1.15 (0.60**–** 2.20)
**Chronic disease status**	
No/don’t know	Reference = 1.0
Yes	2.58 (1.34**–** 4.96)
**Perceived social support**	1.31 (1.12**–** 1.54)
**Active coping**	1.40 (1.10**–** 1.79)
**Self-stigma**	0.80 (0.72**–** 0.88)
**Rumination**	1.75 (1.13**–** 2.70)
**Catastrophizing**	3.12 (1.49**–** 6.51)

*ORc* Crude odds ratio, *CI* confidence interval

^*^Currently unmarried included unmarried, separated/divorced, cohabiting, widowed, and others.

### Structural equation modeling analysis

The confirmatory factor analysis reported satisfactory model fit indices (χ2/df = 42.967/36 = 1.19 < 2; RMSEA 0.03; CFI 0.99; TLI 0.99) for the four latent variables (perceived social support, self-stigma, active coping, and maladaptive emotion regulation). The factor loadings of the latent variables range from 0.68 to 0.98 (all had *p* < 0.05). The results indicated that the measurement model was suitable for the SEM analysis. Information about loadings of these latent variables are presented in Supplementary Fig. 2.

**Fig. 1:**
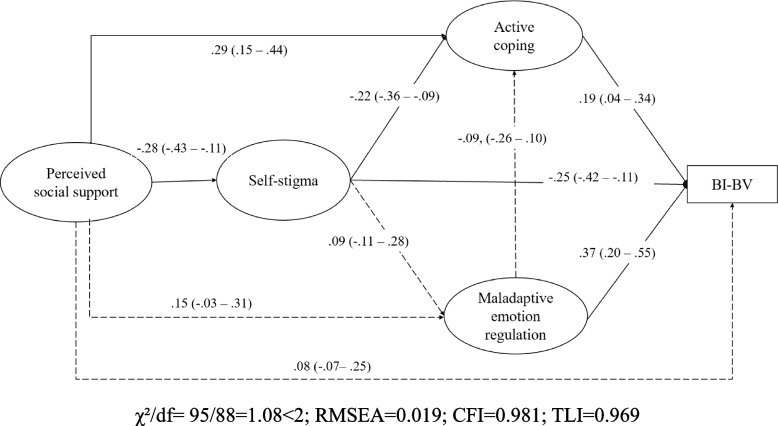
The structural equation model testing the mediation effects. Note: Standardized coefficients are presented. BI-BV = Behavior/intention of taking up booster COVID-19 vaccination

The SEM results are presented in Fig. 1. Background factors were adjusted for. The model yields satisfactory model fit indices (χ^2^/df = 95/88 = 1.08 < 2; RMSEA = 0.019, CFI = 0.981, TLI = 0.969, SRMR = 0.047). Several significant mediations were identified, supporting some of the initial hypotheses. First, the indirect effect of perceived social support on BI-BV via active coping was statistically significant (β = 0.06, 95% CI: 0.02**–**0.12), i.e., perceived social support was positively associated with active coping (β = 0.29, 95% CI: 0.15**–**0.44), which was in turn positively associated with BI-BV (β = 0.19, 95% CI: 0.04**–**0.34). The mediation effect size was 21%. Second, the indirect effect between perceived social support and BI-BV via self-stigma was statistically significant (β = 0.07, 95% CI: 0.03**–** 0.14), i.e., perceived social support was negatively associated with self-stigma (β = −0.28, 95% CI: −0.43**–** −0.11), which was in turn negatively associated with BI-BV (β = −0.25, 95% CI: −0.42**–** −0.11). The mediation effect size was 27%. Third, the serial indirect path of higher perceived social support → lower self-stigma → higher active coping → higher BI-BV was also statistically significant (β = 0.01, 95% CI: 0.002 **–** 0.04), i.e., perceived social support was negatively associated with self-stigma (β = −0.28, 95% CI: −0.43 **–** −0.11), which was in turn negatively associated with active coping (β = −0.22, 95% CI: −0.36 **–** −0.09), which was in turn positively associated with BI-BV (β = 0.19, 95% CI: 0.04**–** 0.34). The mediation effect size was 5%. The total mediation size of the three significant indirect paths was thus 53%. Notably, three other indirect paths, all of which involved emotion regulation as the mediator, were statistically non-significant (perceived social support → maladaptive emotion regulation → BI-BV; perceived social support → self-stigma → maladaptive emotion regulation → BI-BV; perceived social support → self-stigma → maladaptive emotion regulation → active coping → BI-BV). Maladaptive emotion regulation was significantly associated with BI-BV (β = 0.37, 95% CI: 0.20**–** 0.55). Furthermore, the direct effect from perceived social support to BI-BV (β = 0.08, 95% CI: −0.07, 0.25) was statistically non-significant.

## Discussion

This is one of the few studies investigating BI-BV among PECC who completed the primary series of COVID-19 vaccination prior to their diagnosis. To summarize the main findings, the prevalence of BI-BV among PECC was 62.2%. According to the SEM analysis, perceived social support and self-stigma were positively and negatively associated with BI-BV, respectively. Interestingly, the association between perceived social support and BI-BV was fully mediated via 1) self-stigma, 2) active coping, and 3) self-stigma then active coping (a serial mediation). Two of these indirect paths involved the cognitive strategy of active coping. However, the other three mediation indirect paths involving maladaptive emotion regulation were all statistically non-significant. Unexpectedly, in the SEM model, maladaptive emotion regulation was positively associated with BI-BV but was not significantly associated with perceived social support and self-stigma in the SEM.

The suboptimal prevalence of BI-BV observed in this study was lower than the reported prevalence of the primary series vaccination during the pandemic previously recorded in some general populations [66]. Notably, only 19 out of the 230 participants had taken up the vaccination. One reason was that behavioral intention often does not translate into action [67, 68]. Another reason is that as most of the participants were recently infected (within the last six months), it might be too early for them to take up booster vaccination, considering a 6-month interval between diagnosis and vaccination recommended by the professionals. As the pandemic had ended, less people would maintain the health beliefs that are supportive of booster vaccination (e.g., perceived severity of COVID-19), health promotion of vaccination is going to be an uphill challenge. In this study, older and currently married people were more likely than their counterparts to report BI-BV, corroborating previous studies conducted in Hong Kong and other countries [69–71]. Plausible explanations are that older people might have stronger concerns about the consequences of reinfection whereas currently married people might want to protect their spouse and children through vaccination. Furthermore, participants not working full time showed higher BI-BV, plausibly because of their older age and/or having more time to take up vaccination. Health promotion may pay more attention to such groups with lower BI-BV.

A few reasons may partially explain the observed suboptimal prevalence of BI-BV. First, there were no mandate vaccination policies for booster vaccination among PECC in Hong Kong, who thus had no obligation to disclose their booster vaccination status to anyone. Hence, there was no external pressure urging PECC to take up the vaccines although booster vaccination was encouraged by the government and free vaccination was available to all Hong Kong residents at the time of the study. Second, PECC might believe that they had acquired natural immunity that was adequately protective against reinfection [72]. Third, as all the participants were infected with COVID-19 despite completion of the primary vaccination series, some of them might cast doubts on the efficacy of the booster vaccine [14]. In health promotion, PECC should hence be convinced that hybrid immunities resulted from infection and vaccination are important [73] and that the world authorities have recommended booster vaccination for PECC [7–9].

The stress coping model [15] involving cognitive coping (active coping) in terms of BI-BV was well supported by this study, as the indirect paths involving self-stigma and active coping fully mediated the association between perceived social support and BI-BV. The findings suggest that an increase in perceived social support and a reduction in self-stigma which would increase active coping and then BI-BV. The other observed serial mediation involving active coping further supported the stress coping model involving cognitive coping (perceived social support → self-stigma → active coping → BI-BV). To promote BI-BV, it is possible to enhance perceived social support provided by family members, significant others, and co-workers [74]. Self-stigma can be reduced via various interventions, such as psychoeducation, cognitive behavioral therapy, social skills training, etc. [75]. Interventions also need to reduce self-stigma by convincing PECC that COVID-19 infection is not a fault. Active coping can be promoted by using the problem‐solving model [76, 77]. The findings hence have both practical and theoretical significance.

In contrast to the supportive findings regarding the cognitive stress coping model, the mediation paths based on the maladaptive emotion regulation (rumination and catastrophizing) model were all statistically non-significant. Such findings suggest that the cognitive stress coping model worked better than the emotion regulation model in explaining the association between perceived social support and BI-BV. In addition, this study found that active coping was not significantly associated with maladaptive emotion regulation. The two findings suggest that in this case, cognitive coping and emotion regulation are two separate processes. Also, there is no study looking at the effects of coping and emotion regulation jointly in the same model. The findings have thus added knowledge to the discussion comparing the coping and emotion regulation processes. Future studies are warranted to confirm the findings.

Furthermore, there is a dearth of studies investigating the role of emotion regulation in determining booster vaccination among PECC. The only study found in literature reported that rumination was negatively associated with vaccination behavior [78]. In contrast, the latent variable of maladaptive emotion regulation was positively, instead of negatively, associated with BI-BV in this study. An explanation is that rumination/catastrophizing may be seen as either a maladaptive emotion regulation [37] or an expression of fear [79]. The former but not necessarily the latter implies a negative association between maladaptive emotion regulation and BI-BV. According to the fear appeal approach [80], ‘fight’ (removal of the source of fear) or ‘flight’ responses (avoidance of thinking about the situation) may be evoked in a fearful context. Booster vaccination may be seen as a ‘fight response’ to fear, instead of being a maladaptive emotion regulation outcome which should be negatively associated with BI-BV; rumination/catastrophizing may be seen as an expression of fear that initiated a fight response that would lead to vaccination. This perspective may partially explain the ‘unexpected’ positive association between maladaptation and BI-BV. As active coping is an adaptive process, its positive association with BI-BV is expected and vaccination can be interpreted as an adaptive response.

Corroborating previous studies [20, 21, 81, 82] and supporting the initial hypotheses, this study finds that perceived social support and active coping were positively associated with BI-BV and self-stigma was negatively associated with BI-BV. No study has investigated the potential effects of self-stigma due to infection on BI-BV among PECC although a previous study looked at the similar effects in the general population [31]. As COVID-19 affected almost every aspects of life [83], some PECC might have internalized the public stigma, and worried about infecting often or bringing trouble to other people. Stigma and self-stigma regarding infectious diseases (e.g., HIV, SARS, and H1N1) were common [27, 84–86] and were barriers against health service utilization [87]. It is uncertain whether the level of self-stigma has subsided in the post-pandemic period; further research is needed.

A published paper was based on the same dataset that was analyzed in the present paper [14]. It investigated associations between cognitive factors (e.g. perceived susceptibility to COVID-19)/emotional factors (e.g. illness concern) and intention to take COVID-19 booster vaccine among PECC in Hong Kong [14], whereas the present paper looked at the associations between a different set of psychosocial factors (perceived social support and stigma) and behavior/intention of booster vaccination as well as mediators between the associations via active coping and emotional regulation among PECC. The dependent variables of the two papers were different—the current paper looked at both behavior and intention whereas the other paper only looked at intention of booster vaccination. Also, the current but not the former paper included vaccinated PECC in the sample. These two papers thus presented different perspectives. Another study was conducted in Hong Kong from June 20 to July 18, 2022 [88], which investigated multidimensional determinants (e.g., perceptions, information exposure, family influences, and mental distress) of first-dose COVID-19 vaccination behavior among older adults aged 60 years or above in the Hong Kong general population. The current and the other two published papers thus involve different objectives and study populations; they together present a more comprehensive picture about COVID-19 vaccination in Hong Kong.

This study has innovative findings but also several limitations. First, BI-BV might have been over-reported as it is a socially desirable behavior. Second, the moderate response rate of this study might lead to selection bias; characteristics of non-respondents were, however, unavailable for comparisons. Third, a single question was used to assess BI-BV, although similar questions were commonly asked in previous studies [89, 90]. The dependent variable combined vaccination behavior with intention to indicate the overall vaccination tendency after receiving a COVID-19 diagnosis. Fourth, the study did not record the exact duration since diagnosis. Yet, as the survey was conducted from June to August 2022 during the major fifth wave outbreak that peaked after February 2022, the majority of the participants had been diagnosed for less than six months. As vaccination within six months since diagnosis was not recommended to PECC, only 19 participants had taken up a booster vaccination after receiving their COVID-19 diagnosis and were possibly diagnosed during the early phase of the outbreak and interviewed during the late phase of the study period. A sensitivity analysis was conducted by removing such cases and the same findings were obtained. With a larger sample of the two groups, a comparison between factors of intention of booster vaccination between the groups could be conducted. The overall sample size of 230 was not large, but it fits into the rule of thumb for SEM analysis that there should be at least 10 cases per observed variables [91]. Fifth, recall bias was possible; however, its size should be minimum as the recall period was relatively short (< 6 months) and the only question that might be subject to recall bias was about post-diagnosis vaccination (which was a pivotal event) within the last six months. Sixth, temporal or causal inferences are not feasible due to the cross-sectional study design; longitudinal studies are warranted. Seventh, the study was conducted during a major outbreak in Hong Kong, so it is uncertain whether the studied relationships would be stable and valid over time and in the post-pandemic stage. Similarly, as the study was conducted among PECC in Hong Kong, caution should be taken when generalizing the results to other populations. Eighth, the betas of the significant indirect path were relatively small. Researchers have, however, pointed out that indirect effect of such sizes may be seen as ‘small but not negligible’ when it has theoretical/practical implications [92, 93]. Notably, the mediation effect sizes of the three significant indirect paths were 27%, 21%, and 5%; the total mediation size of 53% was substantial; the findings are certainly useful in understanding the mechanisms between perceived social support and BI-BV. Last, there are other unstudied coping strategies apart from active coping. Cautions should be taken when claiming the usefulness of the stress cognitive coping model, as other types of coping strategies may have different effects on BI-BV. Similarly, apart from rumination and catastrophizing, there are also other unstudied maladaptive/adaptive emotion coping responses that might play different roles in explaining BI-BV.

## Conclusions

This study identified significant associations between perceived social support/self-stigma/active coping/maladaptive emotion regulation and BI-BV. Except the unexpected positive association involving maladaptive coping, the other significant associations with BI-BV followed the expected directions. A key finding was that self-stigma and active coping significantly, both individually and serially, mediated between perceived social support and BI-BV. The indirect paths involving emotion regulation were however, all statistically non-significant, signifying that coping (active coping) and maladaptive emotion regulation (rumination/catastrophizing) were two separate processes; the former worked better than the latter in explaining the mechanism between perceived social support and BI-BV. These findings are novel. Interventions are needed to increase BI-BV among PECC by increasing perceived social support, reducing self-stigma, and improving active coping. Reminders should be disseminated to the general public that COVID-19 remains prevalent and harmful whereas booster vaccination is safe and efficacious. Longitudinal studies are needed to verify the findings.

## Supplementary Information

Supplementary Material 1.

Supplementary Material 2.

## Data Availability

The data that support the findings of this study are available from the corresponding author upon reasonable request.

## Abbreviations

BI-BV: Behavior/intention of taking up booster COVID-19 vaccination
PECC: People who have ever contracted COVID-19
RMSEA: Root mean square error of approximation
CFI: Comparative fit index
TLI: Tucker-Lewis index
SEM: Structural equation model
CI: Confidence interval
ORc: Crude odds ratio
df: Degree of freedom
