# Supplementary material for: Perceived social support and self-stigma as factors of COVID-19 booster vaccination behavior and intention via cognitive coping and emotion regulation among people infected with COVID-19 in Hong Kong
Source: BMC Public Health. 2025 Feb 18;25:659. doi: 10.1186/s12889-025-21899-x (PMC11834225; doi:10.1186/s12889-025-21899-x)
Supplement: Supplementary file 1 — Supplementary Material 1. [file 12889_2025_21899_MOESM1_ESM.docx]

**Supplementary Figure 1.** The hypothesized conceptual model based on the stress-coping model


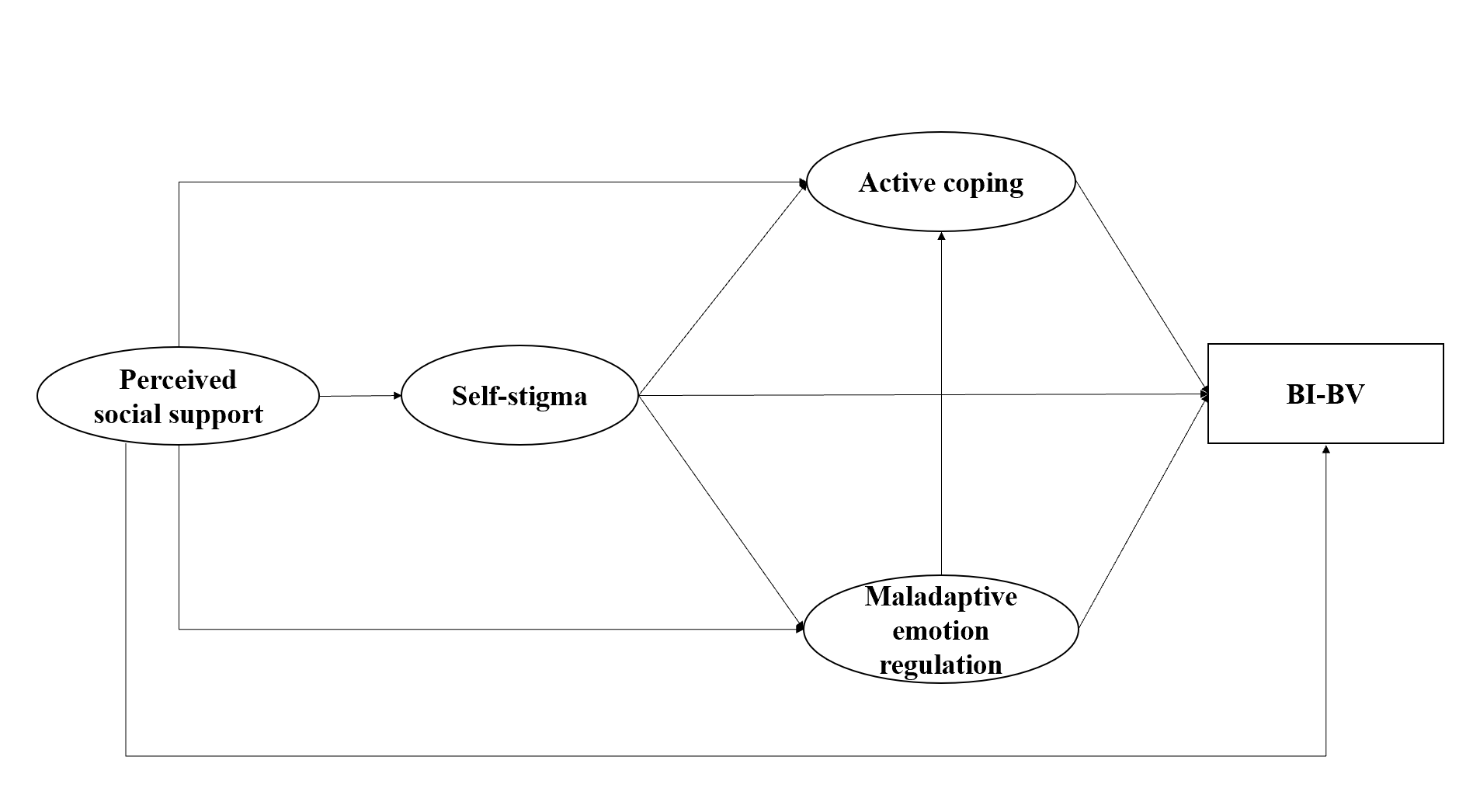


Notes: BI-BV: Behavior/intention of taking up booster COVID-19 vaccination.
