# Supplementary material for: Perceived social support and self-stigma as factors of COVID-19 booster vaccination behavior and intention via cognitive coping and emotion regulation among people infected with COVID-19 in Hong Kong
Source: BMC Public Health. 2025 Feb 18;25:659. doi: 10.1186/s12889-025-21899-x (PMC11834225; doi:10.1186/s12889-025-21899-x)
Supplement: Supplementary file 2 — Supplementary Material 2. [file 12889_2025_21899_MOESM2_ESM.docx]

**Supplementary Figure 2.** Model fit and factor loading of the latent variables


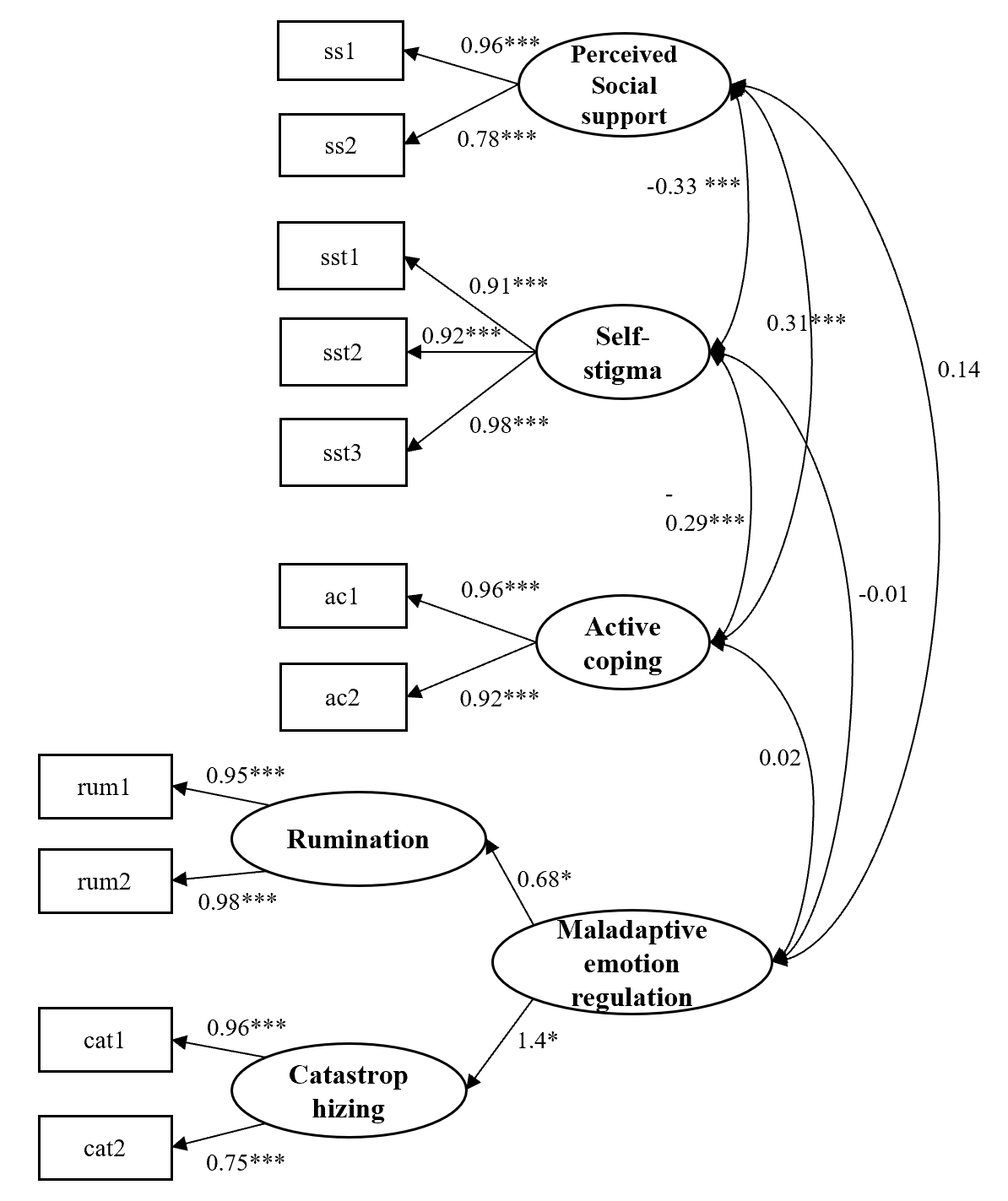


χ2/df = 42.967/36 = 1.19<5; RMSEA=0.03; CFI=0.99; TLI=0.99

**Notes**: Standardized coefficients are presented.

**ss1**: Emotional support, **ss2**: Instrumental support;

**sst1**: Reluctance to disclose COVID-19 infection to others, **sst2**: Perceived negative views from others, **sst3**: Perceived public discrimination;

**ac1**: Concentrating efforts on doing something about the situation,

**ac2**: Taking actions to improve the situation;

**rum1**: Constantly recall the pandemic as well as feelings about the pandemic;

**rum2**: Indulge in thoughts and feelings about the pandemic;

**cat1**: Constantly think about how terrible the pandemic is;

**cat2**: Sometimes feel like the disaster is coming.
